# Scaffold vascularization method using an adipose-derived stem cell (ASC)-seeded scaffold prefabricated with a flow-through pedicle

**DOI:** 10.1186/s13287-019-1535-z

**Published:** 2020-01-23

**Authors:** Tomasz Dębski, Agata Kurzyk, Barbara Ostrowska, Juliusz Wysocki, Jakub Jaroszewicz, Wojciech Święszkowski, Zygmunt Pojda

**Affiliations:** 1 Department of Regenerative Medicine, Maria Sklodowska Curie Institute - Oncology Center, Roentgena 5, 02-781 Warsaw, Poland; 2 Materials Design Division, Faculty of Materials Science and Engineering, Warsaw University of Technology, Woloska 141, 02-507 Warsaw, Poland

**Keywords:** Prefabrication, Flow-through pedicle, Scaffold vascularization, 3D printing, Scaffold, Stem cells, Tissue engineering

## Abstract

**Background:**

Vascularization is important for the clinical application of tissue engineered products. Both adipose-derived stem cells (ASCs) and surgical prefabrication can be used to induce angiogenesis in scaffolds. Our aim was to compare the angiogenic potential of ASC-seeded scaffolds combined with scaffold prefabrication with that of non-seeded, non-prefabricated scaffolds.

**Methods:**

For prefabrication, functional blood vessels were introduced into the scaffold using a flow-through pedicle system. ASCs were isolated from rat fat deposits. Three-dimensional-printed cylindrical poly-ε-caprolactone scaffolds were fabricated by fused deposition modelling. Three groups, each containing six rats, were investigated by using non-seeded, ASC-seeded, and osteogenic induced ASC-seeded scaffolds. In each group, one rat was implanted with two scaffolds in the inguinal region. On the right side, a scaffold was implanted subcutaneously around the inferior epigastric vessels (classic prefabrication group). On the left side, the inferior epigastric vessels were placed inside the prefabricated scaffold in the flow-through pedicle system (flow-through prefabrication group). The vessel density and vascular architecture were examined histopathologically and by μCT imaging, respectively, at 2 months after implantation.

**Results:**

The mean vessel densities were 10- and 5-fold higher in the ASC-seeded and osteogenic induced ASC-seeded scaffolds with flow-through prefabrication, respectively, than in the non-seeded classic prefabricated group (*p* < 0.001). μCT imaging revealed functional vessels within the scaffold.

**Conclusion:**

ASC-seeded scaffolds with prefabrication showed significantly improved scaffold vasculogenesis and could be useful for application to tissue engineering products in the clinical settings.

## Background

One of the greatest challenges facing tissue engineering is ensuring optimal vascularization within tissue engineered products (TEP). Currently, it is possible to create only small tissue substitutes supplied by diffusion such as those utilized in the skin, cartilage, and cornea [1]. To enable the production of other tissues of larger size and even entire organs de novo, it is essential to create a functional vascular network. This vascular network should exhibit fast vascular development and a dense capillary network with a maximum intercapillary distance of 200 μm [2]. In recent years, many strategies for TEP vascularization have been developed. These methods focus on creating an appropriate scaffold structure [3], seeding scaffolds with various cells [4] and growth factors [5], and creating vessels in vitro [6] and in vivo through prefabrication [7]. Although no strategy has been shown to be optimal, prefabrication has shown promising results.

Okuda et al. [8] evaluated the efficacy of a classic prefabrication method (a vascular pedicle located in proximity to the scaffold) and the subcutaneous implantation of a β-tricalcium phosphate scaffold (control group) and found that the average vessel density was threefold higher in the prefabrication group. Additionally, Akita et al. [9] found that prefabrication of a hydroxyapatite scaffold with a ligated vascular pedicle resulted in better scaffold vascularization than subcutaneous implanted scaffolds.

**Fig. 1:**
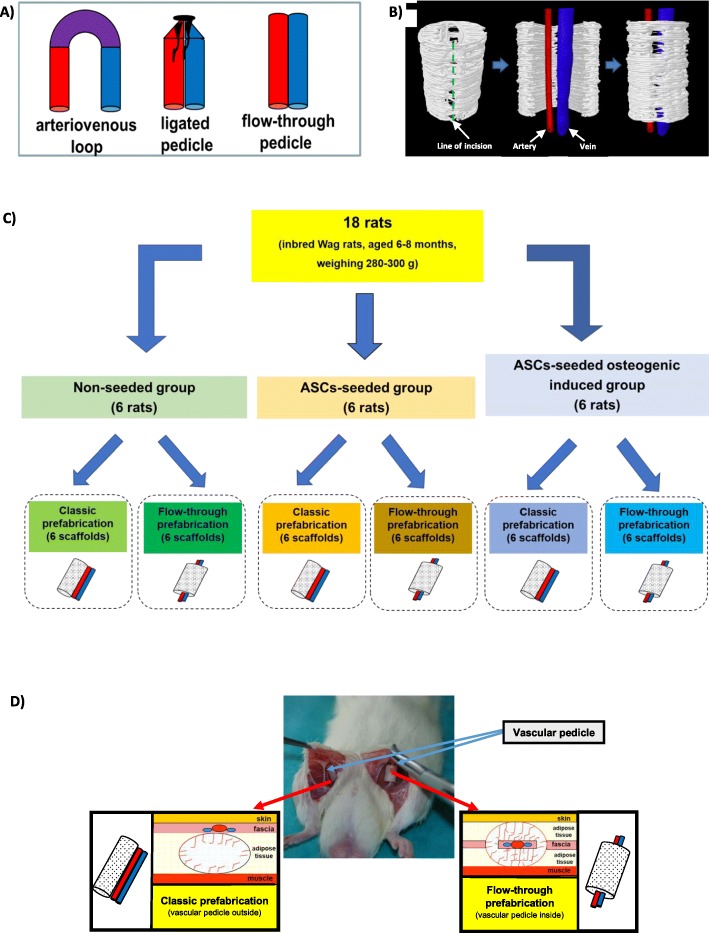
Concept of the flow-through prefabrication method (**a**, **b**) and study design (**c**, **d**): **a** Vascular pedicle types: arteriovenous loop, arteriovenous bundle and flow-through pedicle. **b** New prefabrication method examined by the 3D reconstruction of μCT imaging showing the incision containing the scaffold from one side and its opening and the insertion of the flow-through type vascular pedicle into it. **c** Three groups of six rats each were used: a non-seeded group, an ASC-seeded group and an ASC-seeded osteogenic induced group. **d** Two methods of prefabrication were tested in each rat: classic prefabrication with the vascular pedicle outside the scaffold (vessel ingrowth only from outside) and flow-through prefabrication with the vascular pedicle inside the scaffold with a flow-through pedicle model (vessel ingrowth from outside and inside)

The scaffold prefabrication models tested previously were introduced with a vascular pedicle inside the scaffold after artery and vein anastomosis (arteriovenous loop) [10] or ligation (arteriovenous bundle) [9]. Until now, the prefabrication model involving a flow-through type pedicle located inside the scaffold has not been tested (Fig. 1a). In this model, an intact artery and a vein with a surrounding cuff of tissues was introduced inside a 3D-printed scaffold. Its architecture included an opening on one side to allow the insertion of the pedicle (Fig. 1b).

Additionally, seeding the scaffold with adipose-derived stem cells (ASCs), which have angiogenic properties, has been proposed as an alternative to angiogenic induction in scaffolds [11]. Compared to bone marrow mesenchymal stem cells, ASCs are more accessible, as the total number of stem cells in adipose tissue is approximately 1000-fold higher than that in the bone marrow, and they proliferate sevenfold faster [12–14]. The angiogenic properties of ASCs have been demonstrated in many studies.

Lu et al. [15] showed that the administration of ASCs significantly increased the viability of tissue flaps resulting from the differentiation of ASCs into endothelial cells.

The angiogenic properties of ASCs cultured on special polylactic acid/polyglycolic acid mats were observed by Deng et al. [16].

No studies have evaluated flow-through prefabrication methods using ASC-seeded scaffolds. Therefore, this study was conducted to investigate whether the parallel use of an ASC-seeded scaffold with flow-through prefabrication resulted in greater vessel density compared to the use of non-seeded classic prefabricated scaffolds. Thus, the new method of TEP vascularization investigated in this study is in line with the current trends in the development of efficient methods for scaffold vascularization. This method will thus enable the improved application of TEP in clinical settings. Moreover, it may allow the engineering of vascular tissues that can be independently anastomosed to the host vasculature, or even whole organs called “organoids”. This may lead to major advances in regenerative surgery (TEP for tissue defects) and transplant surgery (organoids for organ transplant).

## Materials and methods

### Study design

All animal experiments were approved by the II Local Bioethical Committee in Warsaw, Poland (Approval number: 31/2011), and performed according to the Guidelines for the Regulation of Animal Experiments.

In the animal model (18 inbred WAG rats, aged 6–8 months, weighing 280–300 g), the effect of scaffold prefabrication was investigated in 3 groups of 6 animals each: a group with non-seeded scaffolds, a group with scaffolds seeded with ASCs, and group with scaffolds seeded with osteogenic induced ASCs (Fig. 1c). In each group, one rat was implanted with 2 scaffolds in the inguinal region. On the right side, a scaffold was implanted subcutaneously in the region of the inferior epigastric vessels by using a classic prefabrication method (classic prefabrication group). On the left side, inferior epigastric vessels were placed inside the scaffold in the flow-through pedicle system by using a new method of prefabrication (flow-through prefabrication group) (Fig. 1d). At 2 months after implantation, the scaffolds were assessed for their effects on angiogenesis by histopathological examination and diagnostic imaging.

### Scaffold fabrication

**Fig. 2:**
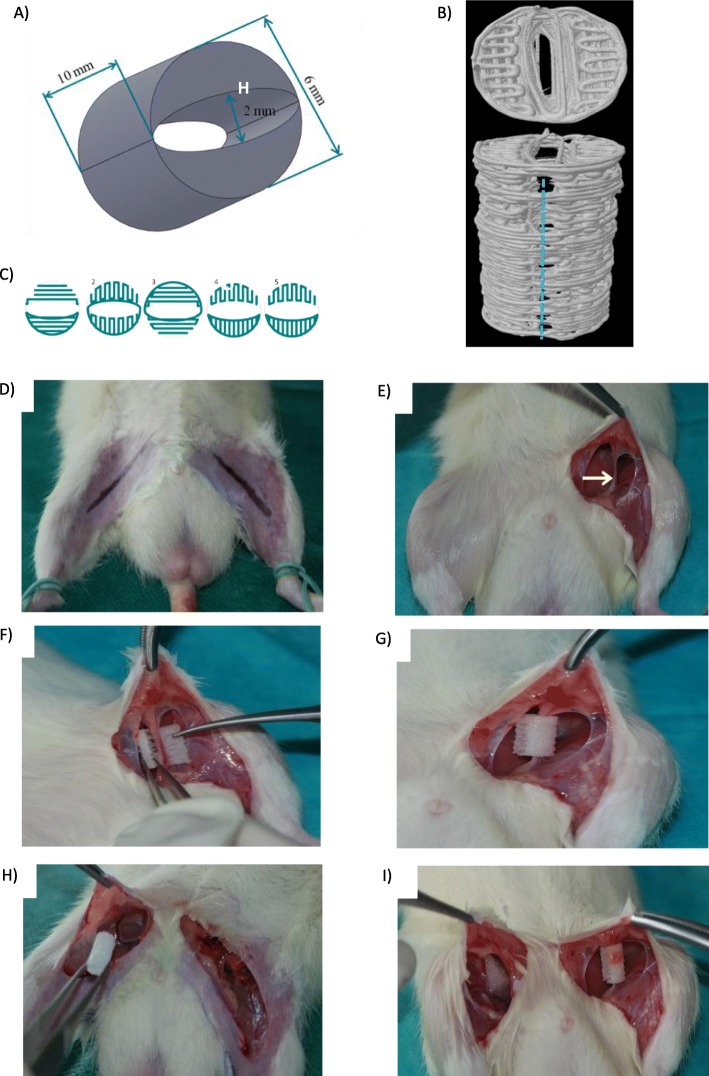
Scaffold design (**a–c**) and scaffold implantation (**d–i**): **a** Scaffold dimensions. **b** Place in the opening of the scaffold (blue line) used to insert the vascular pedicle inside (μCT 3D reconstruction model). **c** The fibre pattern was repeated every five layers during scaffold printing. **d** Incision markings. **e** Isolation of the vascular pedicle with the surrounding fascia. **f** Insertion of the vascular pedicle inside the scaffold. **g** Scaffold “closing” on the vascular pedicle. **h** Classic prefabrication group—insertion of the scaffold in the proximity of the vascular pedicle. **i** Correct placement of scaffolds: on the left side—vascular pedicle is inside the scaffold (flow-through prefabrication group); on the right side, vascular pedicle is outside but close to the scaffold (classic prefabrication group)

A cylindrical scaffold 10 × 6 mm in size with an empty area inside of 6 × 2 mm was designed with *SolidWorks 2012* (Waltham, MA, USA) CAD software (Fig. 2a). The scaffolds were designed to enable the fibers restricting the empty area on one side to be easily cut to allow the scaffold to be opened like a chest to place it on the vascular pedicle (Fig. 2b). Next, 36 poly-ε-caprolactone (PCL) scaffolds were produced with a 3D printer (Bioscaffolder, SYS+ENG, Salzgitter-Bad, Germany) using the fused deposition modelling technique. One scaffold contained 50 layers of fibers, with a fiber pattern that repeated every 5 layers. The fiber pattern used in the first 5 layers is shown in Fig. 2c. Prior to implantation, the scaffolds were sterilized in 70% ethanol.

### Harvesting, isolation, differentiation, and seeding of ADSCs into the scaffold

Fat was harvested from five healthy inbred WAG/W male rats, aged 3–4 months weighing 250–300 g. After the intraperitoneal administration of 200 mg/kg body weight phenobarbital, adipose tissue was harvested from four locations: interscapular, inguinal, gonadal, and perirenal.

The ASCs from the harvested adipose tissues were collected and isolated as described previously [17, 18]. Briefly, the raw adipose tissues were washed extensively with sterile phosphate-buffered saline (Life Technologies, Carlsbad, CA, USA) to eliminate debris and red blood cells. The washed aspirates were treated with 0.075% collagenase (*Clostridium histolyticum*; type I; Sigma-Aldrich, St. Louis, MO, USA) in phosphate-buffered saline for 60 min at 37 °C with gentle agitation. Next, the collagenase was inactivated with 10% foetal bovine serum (Gibco, Grand Island, NY, USA) and the infranatant fluid was centrifuged (400×*g* for 10 min at 23 °C) until phase separation. The stromal vascular fraction pellet was resuspended and passed through a 100-μm filter into a new 50-mL centrifuge tube. The filtrate was centrifuged at 400×*g* for 10 min to obtain the high-density stromal vascular fraction pellet containing the ASCs, which was then cultured in treated tissue culture dishes in medium comprised of equal volumes of low-glucose Dulbecco’s modified Eagle’s medium (Gibco) and foetal bovine serum and incubated in a humidified atmosphere at 37 °C with 5% CO_2_. The media were replaced twice per week, and the cells were passaged upon approaching 80–90% confluence using 0.25% trypsin-EDTA solution (Invitrogen, Carlsbad, CA, USA). After 3–4 passages, the isolated ASCs were seeded onto the sterilized scaffold. Approximately 1 × 10^6^ cells were pipetted onto the upper border of the vertically positioned scaffold and immediately implanted.

To induce osteogenic differentiation, the ASC-seeded scaffolds were incubated in MesenCult™ Osteogenic Stimulatory Supplements (STEMCELL Technologies, Vancouver, Canada). The osteogenic medium contained the following components (all purchased from STEMCELL Technologies): 10^− 4^ M dexamethasone, 1 M β-glycerophosphate, and 10 mg/mL ascorbic acid. The medium was replaced every 3 days for 14 days, after which the scaffolds were immediately implanted.

### Scaffold implantation

Scaffold implantation was performed for 18 healthy inbred WAG male rats aged 3–4 months weighing 280–300 g. After the intramuscular administration of medetomidine (0.2 mg/kg), ketamine (20 mg/kg), and butorphanol (1 mg/kg), the inguinal areas of the rats were epilated and operated under field-sterilized conditions. Next, through a 2.5–3-cm incision in the inguinal area, the superficial epigastric artery with the committing veins was identified and isolated along with 2–3 mm of the surrounding fascia.

On the left side, the PCL scaffold was implanted in a manner that enabled the insertion of unaltered flow-through pedicle (flow-through prefabrication group). On the right side, the scaffold was implanted in proximity to the vascular pedicle, which lay outside the scaffold (classic prefabrication group). The skin was closed with interrupted sutures using Vicryl 4–0 (Fig. 2d–i).

After surgery, the animals were kept under constant observation until they awakened and for another 4 days and were treated with analgesics as needed. After 2 months, the rats were sacrificed by the intraperitoneal administration of 200 mg/kg body weight phenobarbital and the scaffolds were harvested in a manner similar to that used for the implantation. The harvested scaffolds were fixed in 10% buffered formalin and subjected to histopathological analysis.

### Histopathological analysis and histochemical and immunohistochemical staining

The samples from each group were dehydrated using graded ethanol. Clearing was performed using limonene (HistoClear®, National Diagnostics, USA) as a xylene replacement, to avoid the shrinkage of the scaffolds. All samples were embedded in paraffin blocks at 49 °C using low-melting point paraffin. The blocks containing the mid-part of each scaffold (half of the implant height) were serially sectioned into sections of 4 μm thickness using a rotary microtome (Leica RM2245, Leica, Germany). Subsequently, the sections were transferred onto highly adhesive microscope slides (Superfrost Ultra Plus®, Menzel Glaser, Germany). The slides were deparaffinized with limonene and rehydrated.

The slides of each specimen were stained histochemically (HE and von Kossa) and immunohistochemically (CD31and SATB2) to detect the vessels (HE and CD31) and bone formation (von Kossa and SATB2).

After subsequent dehydration, the sections were coverslipped using Canada balsam (Sigma-Aldrich, Munich, Germany) dissolved in limonene as the mounting medium**.**

The slides were stained with haematoxylin and eosin (HE) according to standard procedures (Sigma-Aldrich, Munich, Germany). The HE-stained slides were analysed for the vessel density measurements.

Von Kossa staining was performed to detect any sign of bone formation within the scaffolds. Briefly, the paraffin sections of the scaffolds were decalcified permitting the detection with von Kossa staining of phosphate containing mineralized foci. All samples were fixed with 1.5% paraformaldehyde and paraffin embedded as described above. The sections (4 μm) were deparaffinized, rehydrated and subsequently stained by applying 5% silver nitrate solution under ultraviolet light. The excess silver was removed with 5% sodium thiosulfate. The sections were counterstained with 0.1% nuclear fast red, dehydrated in limonene and mounted as described above. All staining was performed according to the standard staining procedures given by the manufacturer (Silver Plating Kit, Sigma-Aldrich, Munich, Germany).

CD31 endothelial marker staining was performed to confirm the vessel density measured using the HE-stained sections. Briefly, antigen retrieval was performed after the deparaffinization of the sections with a protease solution to avoid the use of a pressure cooker and excessively high temperatures, which could damage the scaffolds. The sections were incubated with Protein Block Serum-Free (Dako, Carpentaria, CA, USA) at room temperature for 10 min to block nonspecific staining. Subsequently, the sections were incubated with antibodies against CD31 (Dako, Carpentaria, CA, USA), overnight at 4 °C. Peroxidase activity was detected using the enzyme substrate DAB (3,3′-diaminobenzidine). For the negative controls, the sections were treated in an identical manner except that they were incubated in Tris-buffered saline without primary antibody. SATB2, a marker of osteoblast differentiation was immunohistochemically stained for the more precise detection of osteogenesis. Briefly, 4-μm sections were automatically pre-treated using the PT-link system (Dako, Glostrup, Denmark) and then stained in an Autostainer Plus (Dako, Glostrup, Denmark) with a monoclonal anti-SATB2 antibody (AAb025742, Atlas Antibodies, Stockholm, Sweden) that was diluted 1: 100.

### Vessel density measurements

The vessel number (*N*) within the scaffold was assessed in both HE-stained and CD31-stained sections. The HE-stained vessels were identified in accordance with generally accepted standards. A vessel was a structure that met two of the following three criteria: it was lined with endothelium, it possessed a well-defined lumen and it contained red blood cells in the lumen.

CD31-positive tube-like structures were identified as vessels in the analysis of the immunohistochemically stained sections. All identified vessels were counted and recorded for each sample.

**Fig. 3:**
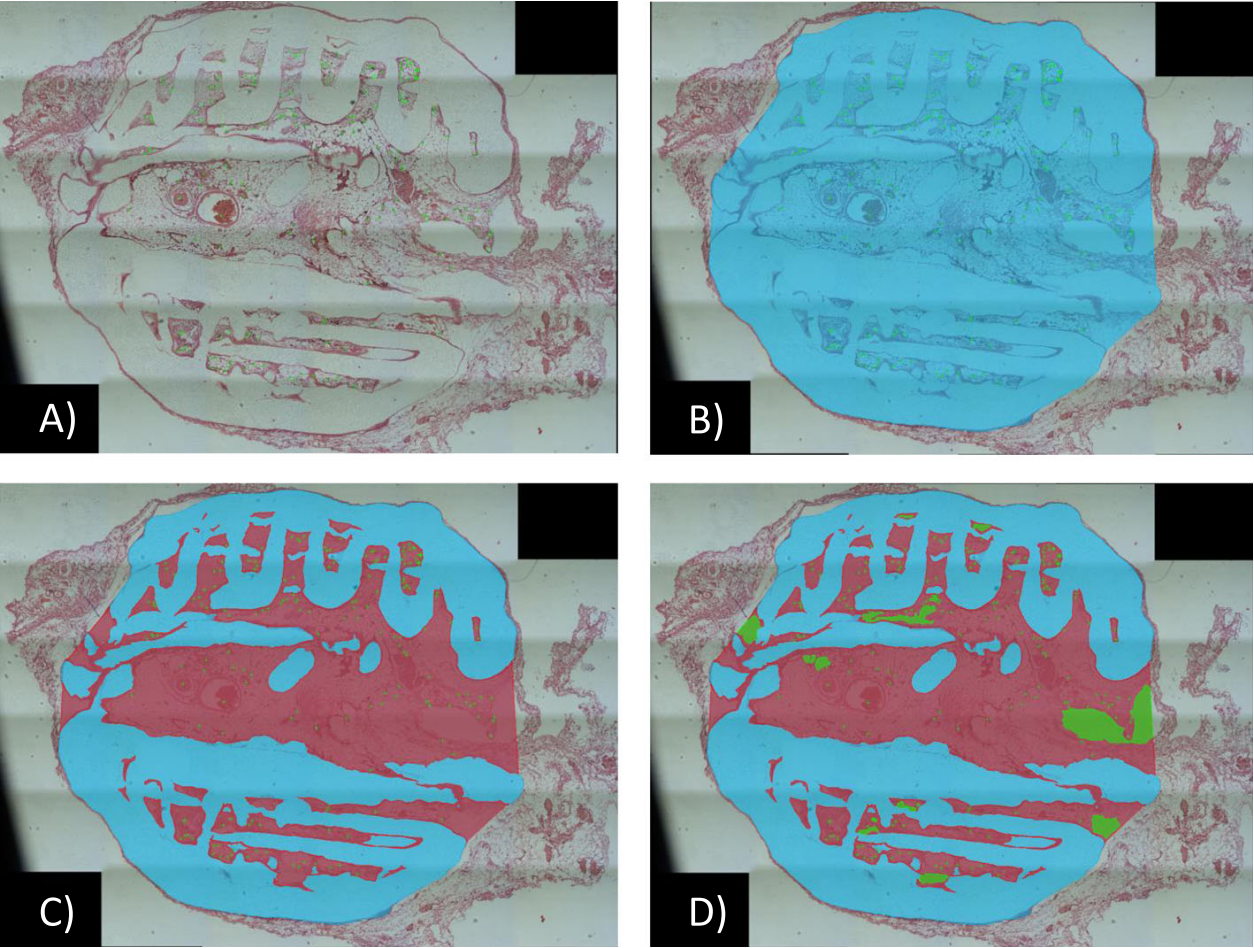
Vascular density analysis: **a** cross-sectional area with the counted number of vessels (N) (green crosses). **b** Specimen cross-sectional surface (S1). **c** Scaffold surface (blue area) (S2). **d** Artefact and empty area surface (green area) (S3)

The prepared and fixed samples were examined using a Nikon Eclipse TI inverted microscope and analysed using digital analysis software (NikonSoftware, Tokyo, Japan). The sample cross sections were analysed to calculate the sample cross-section surface (S1), scaffold surface (S2) and artefacts and empty area surface (exclusion surface (S3) (Fig. 3).

To standardize the results and eliminate errors related to interpretation, the parameter of vessel density was introduced and defined as follows:

$$ \boldsymbol{D}=\frac{\boldsymbol{N}}{\boldsymbol{S}\mathbf{1}-\left(\boldsymbol{S}\mathbf{2}+\boldsymbol{S}\mathbf{3}\right)} $$

where *D* is the vessel density (number of vessels/mm^2^ of tissue), *N* is the vessel number, *S*1 is the sample cross-sectional area, *S*2 is the scaffold surface and *S*3 is the artefacts and empty area surface.

### Exploratory μCT imaging of the scaffold vascularity

A flow-through prefabricated ASCs seeded scaffold and classic prefabricated non-seeded scaffold were explanted from one rat, fixed in 10% formalin, and subjected to contrast imaging as described by Mizutani and Suzuki [19]. After thorough rinsing with demineralized water, the scaffolds were immersed in 50 mL of an iodine solution containing 3% spirit with potassium iodide for 12 h. The scaffolds were rinsed with demineralized water again and placed in a vial containing vegetable oil.

The μCT images were reconstructed using NRecon software (Micro Photonics, Inc., Allentown, PA, USA), and vessels within the scaffold were visualized in 3D using CTVol software (Bruker, Billerica, MA, USA).

In vivo μCT imaging was performed in one rat from the ASC-seeded group using a modified method described by Bolland et al. [20]. Briefly, rats were anaesthetized by the intramuscular injection of medetomidine (0.2 mg/kg), ketamine (20 mg/kg) and butorphanol (1 mg/kg). After epilation and sterilization of the operative field, a midline incision extending across the thorax and abdomen of the rat was made. Abdominal access allowed for the direct visualization of the inferior vena cava into which 0.1 mL of low-molecular weight heparin (2500 IU/mL) was injected. The heart was exposed through a midline sternotomy incision. The left ventricle was cannulated with a 22-G cannula to which a 40-mL syringe containing 0.9% normal saline was attached. A perforation was made in the right atrial appendage and saline infusion was begun. Constant infusion pressure within a physiological range (~ 100 mmHg) was maintained by an infusion pump.

Complete exsanguination was determined by the complete blanching of the liver with clear fluid exiting the right atrial appendage. Next, 40 mL of a contrast solution consisting of iodinated contrast (Ultravist) and dextran 40,000 (0.1 g/mL) at a 1:1 ratio and 2 mL of methylene blue was infused via an infusion pump at a constant sustained rate and pressure (100 mmHg). This was continued until the contrast was visible in the right atrial appendage and within the tail and ear veins. Next, the vascular pedicles were ligated within the scaffolds that had been explanted, fixed with 10% formalin and subjected to μCT scanning.

### Statistical analysis

The vascular density of each of the three groups was determined, and data were statistically analysed using two-way ANOVA with post hoc Tukey test. The data were evaluated using Statistica 13 Software (StatSoft, Inc., Tulsa, OK, USA) and GraphPad Prism 8 (San Diego, CA, USA). The results were considered significant at a probability level of *p* < 0.05.

## Results

All animals included in the study (Fig. 1c, d) survived until the end of surgery (Fig. 2d–i) Two scaffolds were implanted in each rat and prefabricated using classic and flow-through methods (Fig. 1a,b and 2a–c). The rats returned to normal activity within 24 h and experienced an uncomplicated postoperative course until 8 weeks post-surgery. There were no problems related to scaffold implantation, extrusion, infection or rejection. No remarkable resorption was observed macroscopically in all groups at 8 weeks.

### Histological assessments

At retrieval, the scaffolds were completely covered by fibroconnective tissue. No marked inflammatory signs or adverse tissue reactions were found.

**Fig. 4:**
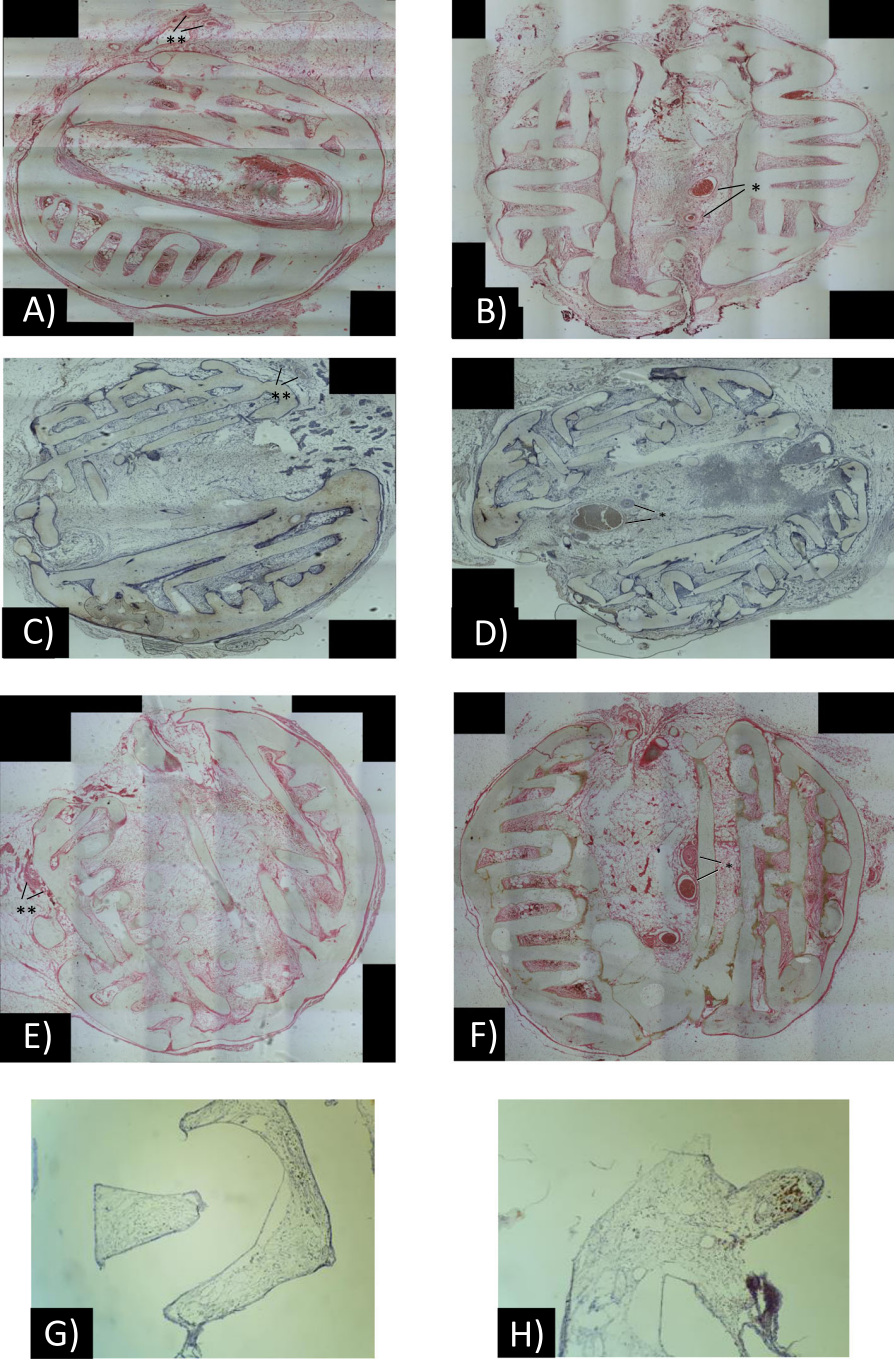
Vessel density analysis was performed using both HE- (**a**, **b**) and CD31-stained(**c**, **d**) sections. The vascular pedicles were visible inside (*) or outside (**) in the flow-through (**b**, **d**) or classic prefabrication group, respectively. Bone formation assessed on von Kossa-stained (**e**, **f**) and SATB2-immmunostained (**g**, **h**) sections was not detected in any groups

HE-stained and CD31-immunostained sections revealed scaffold surrounded by fatty and fibrous tissue. The scaffold architecture was preserved during histopathological preparation, and its parts were surrounded by giant multinucleated histiocytes. In flow-through prefabricated scaffolds, the central part contained large vessels providing dense vascular network to surrounding tissues (Fig. 4b, d). In classic prefabricated scaffolds, the vascular pedicle was outside and vascularity of tissues in the central part of scaffold was lower than in the flow-through prefabricated group (Fig. 4a, b). Von Kossa staining and SATB2 staining did not detect any sign of bone formation and mineralization. Few samples contained multiple hemosiderin-laden macrophages scattered in fatty tissue (Fig. 4e–h).

### Vessel density

To study the effects of scaffold prefabrication and seeding on vascularity, the vessel density was analysed using both HE- and CD31-immunostained sections (Figs. 3 and 4a–d).

Differences in capillary density were observed between the ASC-seeded and flow-through prefabricated group (new method group) and non-seeded and non-prefabricated group (control group). Histological (HE) evaluation indicated that the vessel density, which was an index of neovascularization, was markedly increased in the new method group in both the ASC-seeded and ASC-seeded osteogenic induced scaffolds with values of 21.205 ± 1.543 and 11.715 ± 1.210, respectively, versus the value of 2.182 ± 0.65 in the control group. The capillary density in these groups was approximately 9.7-fold (*p* < 0.001) and 5.3-fold (*p* < 0.001) higher than that in the control group, respectively.

**Fig. 5:**
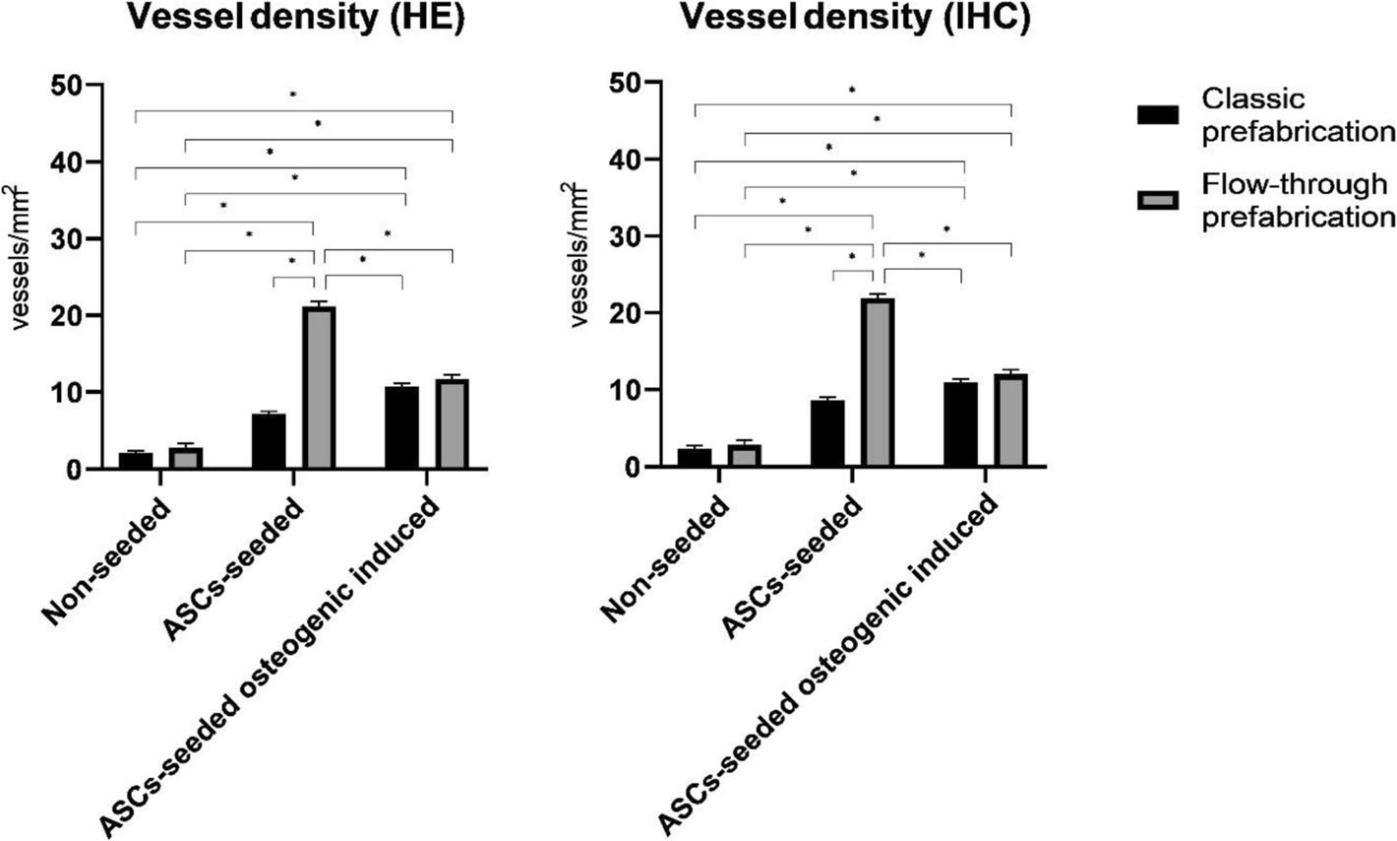
Vessel density assessed on HE-stained (HE) and CD31-stained (IHC) sections. Notes: statistical analysis: * *p* < 0.001

The effect of seeding PCL scaffolds with ASCs was also significant. The capillary density revealed in the cross-sectional analysis of the scaffold was greater in the ASC-seeded and ASC-seeded osteogenic induced groups than in the non-seeded groups. The ASC-seeded flow-through prefabricated and ASC-seeded osteogenic induced flow-through and classic prefabricated groups had significantly (*p* < 0.001) higher capillary density than the corresponding non-seeded flow-through and classic prefabricated groups, with values of 21.205 ± 1.543, 11.715 ± 1.210 and 10.772 ± 0.922 versus 2.792 ± 1.38 and 2.182 ± 0.65, respectively. Although no statistically significant differences were found between the ASC-seeded classic prefabricated group and the non-seeded flow-through and classic prefabricated groups, the capillary density values differed (7.18 ± 0.71 vs. 2.792 ± 1.38 and 2.182 ± 0.65, respectively). The scaffold prefabrication effect on capillary density was also especially noticeable in ASC-seeded group. The capillary density in these groups was approximately threefold increased and statistically significant in the flow-through prefabricated group compared to that in the classic prefabricated group (21.205 ± 1.543 vs. 7.18 ± 0.71 (*p* < 0.001)). These trends were not observed in the ASC-seeded osteogenic induced group, in which the capillary density was slightly greater in the flow-through prefabricated group than that in the classic prefabricated group (11.715 ± 1.210 vs. 10.772 ± 0.922) but was not significantly greater. There were also no significant differences related to the prefabrication type in the non-seeded scaffold group, but we observed a greater capillary density in the flow-through prefabricated group (2.792 ± 1.38 vs. 2.182 ± 0.65) in the classic prefabricated group. These findings were further confirmed by the analysis of immunostained sections, which revealed a slightly higher capillary density in all groups but no influence on the results (Fig. 5, Table 1).

**Table 1:** Vessel density

	Prefabrication type			
	Classic prefabrication		Flow-through prefabrication	
Seeding type	HE	IHC	HE	IHC
Non-seeded	2.18 ± 0.65^d,e,f^	2.36 ± 0.85^d,e,f^	2.79 ± 1.38^d,e,f^	2.85 ± 1.44^d,e,f^
ASC-seeded	7.18 ± 0.71^d^	8.65 ± 0.81^d^	21.20 ± 1.54^a,b,c,d,e^	21.93 ± 1.23^a,b,c,d,e^
ASC-seeded osteogenic induced	10.77 ± 0.92^a,b,d^	10.97 ± 0.8^a,b,d^	11.71 ± 1.21^a,b,d^	12.01 ± 1.31^a,b,d^

All measurements are expressed as the mean ± SEM (*n* = 6)

*HE* hematoxylin and eosin stained sections, *IHC* immunohistochemical stained sections

^a^*p* < 0.001 vs. non-seeded classic prefabricated

^b^*p* < 0.001 vs. non-seeded flow-through prefabricated

^c^*p* < 0.001 vs. ASC-seeded osteogenic induced classic prefabricated

^d^*p* < 0.001 vs. ASC-seeded osteogenic induced flow-through prefabricated

^e^*p* < 0.001 vs. ASC-seeded osteogenic induced classic prefabricated

^f^*p* < 0.001 vs. ASC-seeded osteogenic induced flow-through prefabricated

### Exploratory μCT imaging of the scaffold vascularity

**Fig. 6:**
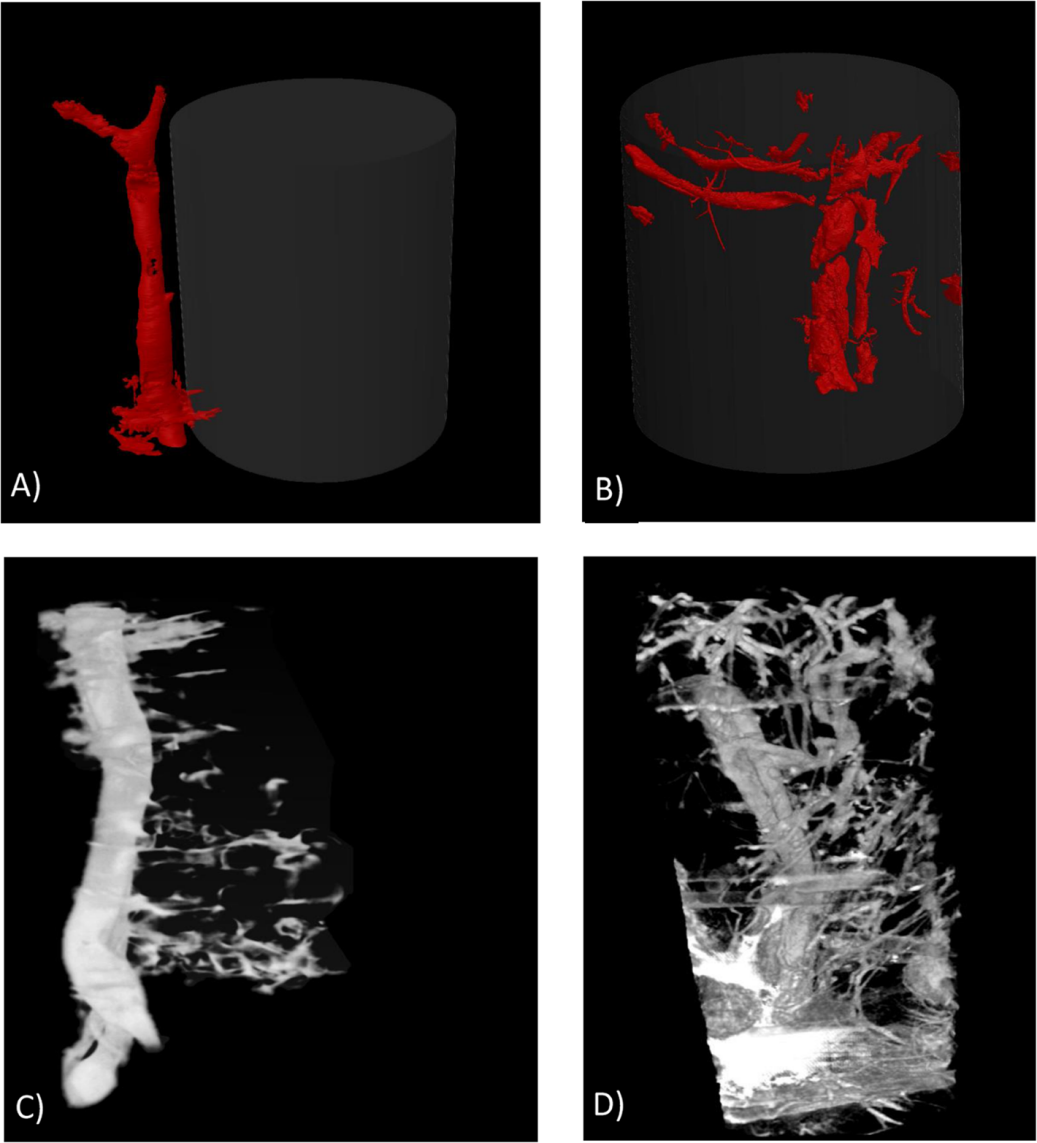
Scaffold vascularity imaging: The classic (**a**) and flow-through prefabrication (**b**) groups were assessed through post-mortem and in vivo μCT imaging (**c**, **d** respectively). Small vessels with perfusion that sprouted from the main vascular pedicle were observed in the flow-through prefabrication group (**b**, **d**). In the classic prefabrication group, vessel ingrowth was noted only on the outer surface of the scaffold adjacent to the vascular pedicle (**a**, **c**)

μCT imaging of samples harvested post-mortem revealed that the vessels created during flow-through prefabrication were fully functional. The presence of contrast agents inside the small vessels revealed the perfusion of the vessels sprouting from the main vascular pedicle (Fig. 6b). Moreover, in vivo analysis showed a dense vascular network sprouting from the main vascular pedicle. Additionally, new vessel ingrowth was observed in the scaffold structure and connected with vessels outside the scaffold (Fig. 6d). In the classic prefabrication group, vessel ingrowth was noted only on the outer surface of the scaffold adjacent to the vascular pedicle (Fig. 6a, c).

## Discussion

The aim of this study was to investigate whether the use of ASC-seeded flow-through prefabricated scaffolds resulted in greater vessel density compared to the use of non-seeded and classic prefabricated scaffolds. The model tested in this study used multiple strategies to induce angiogenesis. It included the fabrication of a scaffold with a suitable structure to facilitate angiogenesis and enable the vascular pedicle to be placed inside, the seeding of the scaffold with ASCs with strong pro-angiogenic properties, and the placement inside the scaffold of a fully capable vascular pedicle with an unstudied configuration (flow-through prefabrication), which facilitated angiogenesis and the remodelling of already existing vessels.

Statistical analysis revealed that the average vessel density obtained with this new prefabrication method was 10-fold higher than that in the control group. Okuda et al. [8] compared the efficacy of the classic prefabrication method (with the vascular pedicle located in proximity to the scaffold) with that of the subcutaneous implantation of the scaffold (control group). Their analysis showed similar correlations to those found in our study. However, the difference was not as significant as that found in the present study, as the average value of the vessel density was only threefold higher than the previous 10-fold increased value. In contrast, there were no differences in the vessel density between the ASC-seeded and ASC-seeded osteogenic induced groups in the previous study, whereas in our study, the ASC-seeded group showed twofold higher vessel density than the ASC-seeded osteogenic induced group. The differences in these results may stem from the different prefabrication methods used by Okuda et al. [8], who used a classic prefabrication method in which the vascular pedicle was placed close to the scaffold rather than using a flow-through pedicle placed inside the scaffold, as was performed in the present study. Okuda et al. [8] used a scaffold composed of β-tricalcium phosphate with a random internal architecture that does not facilitate angiogenesis but has better osteogenic properties than the PCL used to fabricate our scaffolds. This difference in the scaffold material could explain why osteogenesis was not observed in our scaffolds.

The formation of new vessels in the scaffold via prefabrication is achieved mainly by angiogenesis. Angiogenesis involves the formation of new vessels from the pre-existing vascular network by vessel division or sprouting [21].

During this process, endothelial cells are activated and begin to degrade the surrounding matrix by the release of matrix metalloproteinases (MMP-2 and MMP-9). After this, the endothelial cells migrate into the gaps, resulting in the formation of capillary buds and sprouts. Endothelial cells that are located behind the migrating endothelium proliferate, thereby elongating the newly developing blood vessel [22]. Vessels form small capillaries that, through the process of arteriogenesis, develop into arteries and veins [23].

After the implantation of the scaffold, angiogenesis occurs spontaneously. This is in part due to an inflammatory wound healing response induced by the surgical procedure. Furthermore, the seeded cells create a hypoxic state in the scaffold, which stimulates the endogenous release of angiogenic growth factors and chemokines [22].

One of the key angiogenic growth factors is vascular endothelial growth factor (VEGF), which is activated by hypoxia-inducible factor 1 (HIF-1) in an ischaemic microenvironment [24]. VEGF is responsible for the proliferation, migration, differentiation and survival of endothelial cells [25]. During the sprouting of endothelial cells, endothelial tip cells migrate towards the hypoxic stimulus while proliferating stalk cells follow behind and elongate the newly formed vessels. A VEGF gradient regulates the shape of the newly formed vessel by guiding tip cell migration. In addition, this induces the process of vessel elongation and lumen formation [26, 27].

Basic fibroblast growth factor (bFGF) is another important pro-angiogenic growth factor that participates in the initiation of the migration and proliferation and differentiation of endothelial cells and, through its interaction with smooth muscles, is involved in the structural enlargement of developing vessels [28].

Platelet-derived growth factor (PDGF) is also involved in the maturation and remodelling of vessels through the recruitment of mural cells, i.e. smooth muscle cells and pericytes [29].

All of these pro-angiogenic growth factors can be secreted by ASCs in a hypoxic environment. Yang et al. [30] revealed that ASCs function in angiogenesis via the paracrine secretion of growth factors, such as VEGF, bFGF, transforming growth factor (TGF-β1, TGF-β2), hepatocyte growth factor, PDGF-AA and others. Recent studies have also demonstrated the ability of ADSCs to secrete membrane-derived microvesicles (MVs) containing pro-angiogenic particles, such as FGF2, PDGF, VEGF, and matrix metalloproteinases 2 and 9 [31, 32].

All of these growth factors and pro-angiogenic particles released by ASCs play an important role in angiogenesis as described above.

The development of new sprouts from pre-existing vessels may explain the synergistic effects of the new flow-through prefabrication method and the secretion by ASCs of angiogenic growth factors. Because the ASCs used in the experiment were isolated from adipose tissue and passaged four times, the cells used to seed the scaffolds likely did not include endothelial progenitor cells with confirmed angiogenic properties. Therefore, these results reveal the significant role of ASCs in angiogenesis. Similar conclusions were reached by researchers studying the angiogenic properties of ASCs in ischaemic tissues [33].

Lu et al. [15] showed that the administration of ASCs significantly increased the viability of tissue flaps resulting from the differentiation of ASCs into endothelial cells. They collected ASCs from adipose tissue in the inguinal region of mice, and after three passages that were followed by labelling, the cells were administered to the elevated skin flaps. The control groups were administered medium without ASCs but with mature adipocytes and bFGF. After a 7-day follow-up, the group that was administered ASCs showed higher flap survival, a larger number of vessels and the presence of stain used to label ASCs in endothelial cells.

Koike et al. [34] seeded endothelial cells with MSCs on a gel composed of type I collagen and fibronectin and found a higher number of vessels with increased viability (over a year) than that found in a culture of endothelial cells alone. The vessels that developed in the gel seeded with endothelial cells only were immature and did not function (no perfusion). These results show that ASCs play a role not only in the formation, but also in the stabilization and maturation of newly formed vessels.

Thus, the function of stem cells is not only to deliver the cells required to form vessels, but also to deliver the required growth factors and regulate the process. Indeed, the ability of ASCs to differentiate into endothelial cells and regulate this process via paracrine secretion, combined with the presence of mechanically introduced vessels primed for angiogenesis inside the scaffold, improved angiogenesis in the present study. In addition to the vessels constituting the pedicle, a tissue cuff (fascia) exists around these vessels with numerous capillaries that could undergo sprouting and/or arterialization.

The close proximity of undamaged vessels located inside the scaffold (the proposed flow-through pedicle) and the presence of a large number of ASCs surrounding these vessels (ASCs seeded scaffold) jointly lead to increased effectiveness of angiogenesis, which was shown in the present study.

Unexpectedly, in the ASCs-osteogenic induced group, bone formation was not observed.

**Fig. 7:**
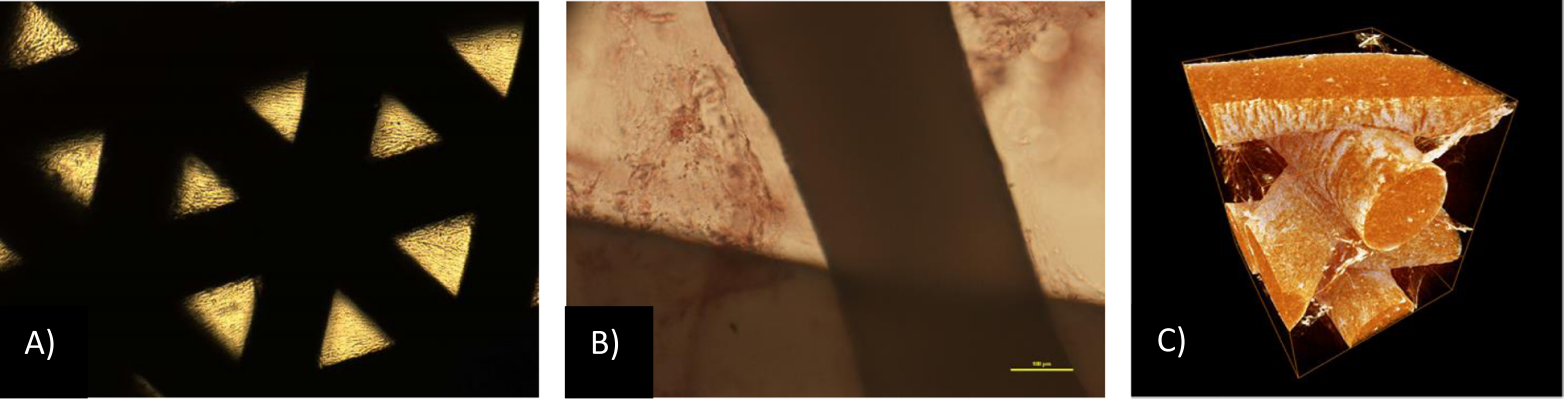
In vitro studies on ASCs seeded PCL scaffolds: **a**, **b** Representative images of ASCs seeded into PCL after 21 days of culture. Alizarin red staining and mineralization assays of rat ASCs after 21 days: **a** control, non-differentiation culture of rat ASCs, **b** osteogenic differentiation. Seeding density of ASCs: 0.9 × 10^6^/scaffold. Representative images are shown at × 10 magnification. Scale bars represent 100 μm. Images were obtained using an Olympus CKX41 microscope. **c** Typical μCT images of the PCL and scaffolds seeded with ASCs and incubated for 21 days. The images were reconstructed and analysed using the SkyScan NRecon and CtAn software. The 3D images were generated using SkyScan CTvol

Interestingly, our previous work [18] in an animal model showed that ASCs seeded into PCL and PCL + 5% TCP promoted proliferation, adhesion and osteogenic differentiation. The osteogenic assay showed that ASCs seeded on the PCL scaffold that were cultured in osteogenic medium presented significantly higher alkaline phosphatase levels during a 14-day culture period compared with ASCs cultured in the control medium (undifferentiated ASCs). These results were also confirmed by Alizarin Red staining (Fig. 7). We also noticed that the scaffolds promoted the proliferation and adhesion of ASCs. We observed that seeded ASCs adhered to the surface and migrated into scaffolds that were cultured for up to 21 days, as confirmed by DAPI staining. Moreover, the metabolic activity assessed via the MTS proliferation assay demonstrated the increase in proliferation after the 21st day of culture of ASCs on PCL [18]. However, the complete molecular process of ASC differentiation during osteogenesis and the mechanism of their cell-specific uptake remains to be fully characterized in future studies. Many studies have confirmed that ASCs can transform into osteoblast-like cells after osteogenic induction in vitro, but only a limited number of studies have investigated whether the osteogenic induction of ASCs can enhance their osteogenic ability in vivo.

In our study, the lack of bone tissue within the scaffold may have been caused by the low osteogenic induction properties of PCL, insufficient osteogenic induction by bone morphogenetic proteins (BMPs) and other osteogenic growth factors in the in vivo conditions [35] and the decreased osteogenic potential of ASCs compared to that of bone marrow mesenchymal stem cells [36]. Thus, this warrants further investigation and is a limitation of this study.

Another limitation was the small number of animals in each group.

Surprisingly, in the ASC-seeded-osteogenic induced group, the vessel density was higher than that in the non-seeded group, demonstrating that after differentiation, the angiogenic potential of ASCs was preserved. Additional studies are needed to clarify the specifics of these effects.

Based on our results, we predict that to achieve the rapid and efficient formation of a vascular network in ischaemic tissues, such as in tissue flaps affected by necrosis or in ischaemic limbs or digits, it is necessary to administer stem cells to the site where the main vessel supplying a given area (vascular pedicle) is located. The results of the present study indicate that new vessel formation occurs much more rapidly if stem cells are in proximity to nutritive vessels. Although the angiogenic properties of stem cells were confirmed previously in such cases [15, 33], their site of administration was random. The administration of stem cells directly into the area of the vessel supplying the necrotizing flap or ischaemic limb or digit will lead to the more rapid formation of a dense vascular network compared to the administration of these cells into randomly chosen area, such as the area that is most ischaemic. The treatment of ischaemia in the early stages is a key factor determining the survival of ischaemic tissues. Further studies using a model of tissue ischaemia are necessary to confirm the efficacy of this method in treating ischaemic tissues.

## Conclusions

Our new method of prefabrication ensures the much faster and greater development of a vascular network within a TEP compared to the classic method. The presence of adipose-derived stem cells within the scaffold and scaffold prefabrication using the new method had synergistic effects (10-fold increase in vessel density versus that in the control). Moreover, the strong angiogenic effect of adipose-derived stem cells was observed following their administration to the area containing the large nutritive vessels and administration at this site could be useful for treating ischaemic tissues.

## Data Availability

The datasets used and/or analysed during the current study are available from the corresponding author on reasonable request.

## Abbreviations

ASCs: Adipose-derived stem cells
bFGF: Basic fibroblast growth factor
BMP: Bone morphogenetic protein
DAPI: 4′,6-Diamidino-2-phenylindole
MMP: Matrix metalloproteinases
MVs: Microvesicles
PCL: Poly-ε-caprolactone
PDGF: Platelet-derived growth factor
TCP: Tricalcium phosphate
TEP: Tissue engineered products
TGF: Transforming growth factor
VEGF: Vascular endothelial growth factor
